# Simulated brushing effects on surface roughness and color stability of coated additively manufactured dental resin: an in vitro study

**DOI:** 10.25122/jml-2025-0181

**Published:** 2026-01

**Authors:** Zahra Alsalem, Mohammed Alothemeen, Daliah Alshehri, Faisal Masaud, Yazeed Alshehri, Lamya ALSalem, Sarah Mohammed Alghamdi, Raghad Saeed Alqahtani, Hussain IbnAhmad

**Affiliations:** 1 Dental department, private practice, Dammam, Saudi Arabia; 2 Dental department, private practice, Abha, Saudi Arabia; 3 College of Dentistry, Imam Abdulrahman Bin Faisal University, Dammam, Saudi Arabia; 4 Dammam Medical Complex, Eastern Province, Saudi Arabia

**Keywords:** 3d printed resin, color stability, dental materials, surface roughness, temporary restorations

## Abstract

This study aimed to evaluate the effect of a glaze coating on the surface roughness and color stability of 3D-printed temporary crown resin materials before and after simulated toothbrushing. Nano-filled ceramic resin specimens were fabricated using a 3D printer (Dentafab) with standardized dimensions of 10 mm in diameter and 2 mm in thickness. The specimens were divided into two groups: uncoated specimens serving as the control group and specimens coated with Optiglaze. Surface roughness and color measurements were recorded at baseline and after brushing simulation. Statistical analysis was performed using two-way analysis of variance (ANOVA) and paired-samples *t*-tests with StatPlus and JASP software, with the level of significance set at *P* < 0.05. Specimens treated with the glaze coating exhibited lower surface roughness values than the uncoated control group. However, no statistically significant differences were observed within either group before and after brushing. Additionally, simulated brushing did not result in clinically perceptible color changes in either group. Applying a glaze coating to 3D-printed temporary crown resin materials improves surface smoothness while maintaining color stability, even after simulated toothbrushing.

## Introduction

The advancement of three-dimensional (3D) printing technology has led to significant progress in restorative dentistry. Additive manufacturing enables the layer-by-layer fabrication of complex dental restorations with high precision, minimal material waste, and a high degree of customization, distinguishing it from conventional subtractive (milling) techniques [1]. As a result, 3D printing has become increasingly integrated into digital dental workflows, offering particular advantages for interim restorations, including streamlined production, reduced fabrication time, and seamless compatibility with digital design systems [2].

In prosthodontics, provisional crowns play a critical role in protecting prepared teeth, maintaining occlusal stability, preserving esthetics, and supporting periodontal health during the interval before definitive restoration placement. These interim restorations are commonly fabricated using ceramic or resin-based materials [3]. Ceramic provisional restorations typically demonstrate excellent esthetics, translucency, and surface stability; however, they are associated with higher costs, longer fabrication times, and limited practicality for short-term clinical use.

In contrast, 3D-printed resin materials provide a cost-effective and efficient alternative, offering acceptable mechanical performance and esthetic properties suitable for temporization. Nevertheless, resin-based materials are generally more susceptible to surface degradation, including increased surface roughness, wear, and color alteration, when exposed to intraoral mechanical and chemical challenges such as toothbrushing, dietary staining, and saliva, compared with ceramic materials [4].

To enhance the surface smoothness, gloss, and color stability of resin-based provisional restorations, various glazing and surface-coating agents have been developed. These coatings aim to produce smoother, glossier surfaces, thereby reducing plaque accumulation and serving as protective barriers against staining and mechanical wear [5]. Previous studies have reported that applying coating agents to resin-based composites can significantly improve surface smoothness and reduce roughness after simulated toothbrushing [6]. By sealing micro-irregularities inherent to 3D-printed surfaces, glazing or coating procedures may improve esthetic appearance and extend the functional longevity of resin restorations. However, the durability of these coatings under repeated mechanical stress, such as toothbrushing, remains a critical factor influencing long-term clinical performance and warrants further investigation.

Toothbrushing simulation is widely employed in dental materials research to replicate the mechanical wear associated with daily oral hygiene practices. Previous investigations have demonstrated that repeated brushing may lead to increased surface roughness, reduced gloss, and measurable color changes in resin materials and resin-based composites [7]. A systematic review reported that most resin-containing CAD/CAM materials exhibited increased surface roughness following simulated toothbrushing [8]. Similarly, in vitro studies have shown that brushing simulation significantly affects the surface roughness and gloss of resin composites [7]. However, limited data are available on the surface and optical properties of recently introduced 3D-printed temporary nano-filled ceramic resins, underscoring the need for further investigation.

Therefore, this study aimed to evaluate the effect of simulated toothbrushing on the surface roughness and color stability of coated and non-coated 3D-printed temporary crown resin materials. The null hypothesis was that no significant differences would be observed between coated and non-coated specimens in surface roughness and color stability, before or after the brushing simulation.

## Material and methods

### Study design and materials

This in vitro study aimed to evaluate the effect of simulated toothbrushing on the mechanical and physical properties of a 3D-printed nanofilled ceramic resin. In the present study, a DentaFab SEGA Pro DLP-based dental 3D printer (3BFAB Technology Inc., China) was used to fabricate specimens from a nano-filled ceramic crown resin material (Dentafab Power Resins). Surface coating was applied using OptiGlaze (GC America, Inc.).

Brushing simulation was performed using a commercially available dentifrice (Colgate Total) and a soft-bristle toothbrush (Colgate Twister; Colgate-Palmolive, São Paulo, SP, Brazil).

### Sample preparation

Disc-shaped specimens (10 mm in diameter and 2 mm in thickness) were digitally designed and exported as STL files, then imported into the 3D printer. A total of 18 disc-shaped specimens were printed from Dentafab ceramic resin using the Dentafab printer at a 0-degree orientation with a layer thickness of 50 µm. For the post-printing process, the unpolymerized resin was removed with 99.9% isopropyl alcohol (Saudi Pharmaceutical Industries, Riyadh, KSA), followed by post-curing at 60°C with a wavelength of 405 nm. Diamond discs were used to remove the support structures, and silicon carbide grinding papers (up to 3000 grit) were used to finish and polish the specimens. The specimens were divided into two main groups (*n* = 9 per group). The first group served as the control group (uncoated), while the second group was coated with OptiGlaze. The coating was applied by placing 30 µL of resin coating onto the specimen surface. A celluloid matrix strip was positioned over the surface prior to light curing to ensure uniform distribution of the coating layer.

### Surface roughness measurements

After specimen preparation, baseline surface roughness measurements were obtained using a non-contact optical profilometer (Contour GT-K 3D Optical Profiler, Bruker Nano GmbH, Berlin, Germany). Initial measurements were recorded after polishing, and post-brushing measurements were obtained following the brushing simulation. A scanning speed of 0.5 mm/s and a cutoff value of 0.8 mm were used. The mean surface roughness (Ra) value was calculated from the recorded measurements [9].

### Color assessment

Color change (ΔE) was evaluated using a spectrophotometer (Color-Eye® 7000A; X-Rite, Grand Rapids, USA). The CIE L*a*b* color system was used to assess color variation between two measurement points using the following formula:

ΔE = [(ΔL)^2^ + (Δa)^2^ + (Δb)^2^]^1/2^

where:

ΔL represents the difference in lightness, Δa represents the difference along the red–green axis, and Δb represents the difference along the yellow–blue axis.

The spectrophotometer was calibrated according to the manufacturer’s instructions. Each specimen was positioned against the measurement port, and the support arm was locked to ensure stability. Color measurements were recorded for each specimen, and mean ΔL, Δa, and Δb values were calculated. Measurements were performed with a 10 mm aperture and D/8° (diffuse/8°) geometry, using a black background. Each specimen was measured twice before and after the brushing simulation [10].

The ΔE values were converted to National Bureau of Standards (NBS) units (trace = 0.0–0.5, slight = 0.5–1.5, noticeable = 1.5–3.0, appreciable = 3.0–6.0, much = 6.0–12.0, and very much > 12) using the following formula.

### Brushing simulation

After baseline measurements, all specimens were mounted in a putty index holder and subjected to simulated toothbrushing using a toothbrushing machine (Model ZM-3.8; SD Mechatronik GmbH, Feldkirchen-Westerham, Germany). The simulation consisted of 20,000 brushing strokes, representing approximately two years of clinical toothbrushing, applied at a rate of 75 strokes per 60 seconds for a total duration of approximately 2 hours and 22 minutes, under a constant load of 0.2 N.

The brushing slurry was prepared by mixing 250 g of toothpaste with 1 L of distilled water, following ISO 11609:2010 guidelines. After brushing, specimens were rinsed with distilled water, and surface roughness and color measurements were repeated [11-15].

### Statistical analysis

Statistical analyses were conducted to evaluate the effects of coating application and brushing simulation on surface roughness (Ra) and color change (ΔE). Descriptive statistics, including mean, median, standard deviation, minimum, and maximum values, were calculated for all groups.

A two-way analysis of variance (ANOVA) was performed in StatPlus to assess the main and interaction effects of coating condition (coated vs. uncoated) and brushing condition (before vs. after) on surface roughness. Additional analyses were performed using JASP software. Paired-samples *t*-tests were used to compare pre- and post-brushing values within each group for both surface roughness and color change. Statistical significance was set at *P* < 0.05 for all analyses.

## Results

**Figure 1:**
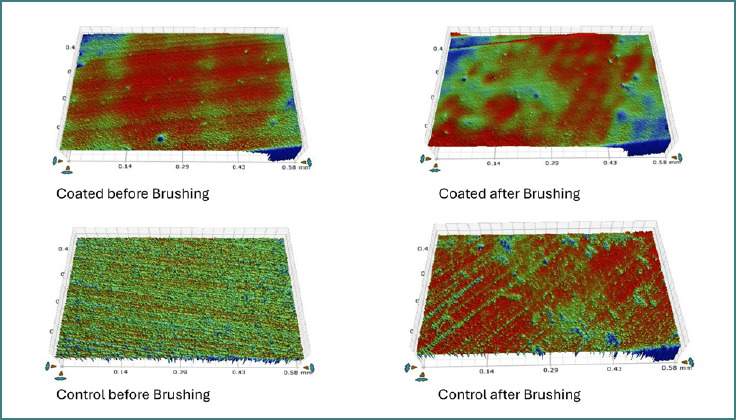
Surface roughness (Ra) measurements of both coated and non-coated groups before and after the brushing simulation

**Figure 2:**
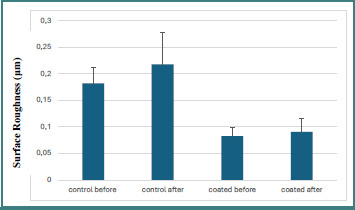
Surface roughness (Ra) values of coated and uncoated specimens before and after brushing represented with standard deviation and mean values

The descriptive surface roughness (Ra) data demonstrated that coated specimens consistently exhibited smoother surfaces than non-coated controls both before and after brushing (Table 1, Figures 1-2). Prior to brushing, the coated group showed a mean Ra value of 0.08252 μm, which increased slightly to 0.09023 μm after brushing. In contrast, the non-coated control group showed notably higher roughness values, with a mean of 0.18204 μm before brushing and 0.21733 μm after brushing. However, brushing resulted in a minor increase in roughness for both groups.

**Table 1:** Descriptive statistics of surface roughness (Ra) values for coated and non-coated specimens before and after brushing

Groups	Sample size	Sum	Variance	Std Dev	Mean	95% Confidence Interval*	
Coated after brushing	9	0.81207	0.00064	0.02528	0.09023	0.07080	0.10966
Coated before brushing	9	0.74270	0.00025	0.01582	0.08252	0.07036	0.09468
Control after brushing	9	1.95600	0.00372	0.06103	0.21733	0.17042	0.26425
Control before brushing	9	1.63833	0.00085	0.02911	0.18204	0.15966	0.20441

A two-way ANOVA was conducted to assess the independent and combined effects of coating application and brushing procedure on surface roughness (Ra) (Table 2). The analysis revealed no statistically significant main effect for coating (*P* = 4.85453E-10), indicating that although coated specimens demonstrated numerically lower roughness values, the difference did not reach statistical significance. Similarly, the brushing factor showed no significant effect on Ra values (*P* = 0.15670), confirming that brushing did not produce a measurable change in roughness across both coated and non-coated groups. Furthermore, no significant interaction between the two factors was observed, suggesting that coating presence did not influence the response to brushing.

**Figure 3:**
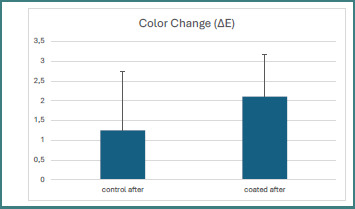
Mean color change (ΔE) values of coated and uncoated specimens after brushing, with mean values and standard deviation

A paired-samples *t*-test was performed to assess changes in surface roughness (Ra) within the coated and non-coated groups before and after brushing, and to compare color change (ΔE) values between the two groups after brushing (Table 3). In the coated group, simulated brushing did not result in a statistically significant change in surface roughness (*P* = 0.365), indicating that the coating layer effectively preserved surface integrity and limited texture alteration following brushing. In contrast, the non-coated control group exhibited a greater numerical increase in surface roughness after brushing (*P* = 0.057). Although this difference did not reach statistical significance (*P* < 0.05), it suggests a tendency toward increased surface deterioration in the absence of coating protection. Regarding color stability, no statistically significant difference in color change (ΔE) was observed between coated and non-coated specimens after brushing (*P* = 0.295). Despite this, coated specimens showed a numerically lower color change than the control group, suggesting a potential protective effect of the coating against discoloration, although the magnitude of this effect was insufficient to achieve statistical significance. Figure 3 presents the difference between the control and coated groups after brushing relative to baseline measurements and shows that all changes were within the acceptable clinical range. The control group showed less color change after brushing, while the coated group showed greater color change than baseline.

**Table 2:** Two-way ANOVA results for the effect of coating and brushing on surface roughness (Ra)

Source of Variation	SS	d.f.	MS	F	*P* value
Factor #1 (Coated)	0.11150	1	0.11150	79.12905	4.85453E-10
Factor #2 (Before)	0.00297	1	0.00297	2.10670	0.15670
Factor #1 + #2 (Coated x Before)	0.00287	1	0.00287	2.03608	0.16360
Within Groups	0.04368	31	0.00141		
Total	0.16103	34	0.00474		

**Table 3:** Paired samples *t*-test results for surface roughness (Ra) within coated and non-coated specimens before and after brushing, and for color change (ΔE) between coated and non-coated specimens after brushing

Parameter	Groups	(Mean ± SD)	After brushing (Mean ± SD)	*t*	df	*P* value
Surface roughness	Coated (before vs after) brushing	0.083 (±0.016)	0.090 (±0.025)	-0.961	8	.365
(Ra)	Control (before vs after) brushing	0.182(±0.029)	0.217(±0.061)	-2.223	8	.057
Color change (ΔE)	Coated vs Control (All after brushing)	2.10530(±1.07137)	1.24586(±1.48821)	-1.119	8	.295

## Discussion

Provisional restorations represent a critical phase in fixed prosthodontic treatment, particularly when immediate placement of the definitive prosthesis is not feasible. The use of 3D-printed provisional crowns has recently gained popularity due to advancements in digital dentistry and additive manufacturing technologies [1]. The present study aimed to evaluate the effect of simulated toothbrushing on the surface roughness and color stability of coated and non-coated 3D-printed resin materials used for provisional restorations.

The results of the current study demonstrated a reduction in surface roughness in coated specimens compared with non-coated samples. These findings are consistent with previous studies evaluating the effect of glazing techniques on ceramic surfaces, which reported that the application of powder-and-liquid glaze materials effectively reduced surface roughness [15]. The observed improvement in surface smoothness can be attributed to the formation of a continuous glaze layer that fills surface irregularities and minimizes micro-defects.

The present study also revealed no statistically significant differences within the groups following the brushing simulation. However, slight increases in surface roughness were observed in both coated and non-coated groups after brushing. Similar observations were reported in a previous study evaluating the effect of simulated toothbrushing on the surface roughness of 3D-printed materials, which demonstrated noticeable increases in roughness after brushing [16]. In contrast, ceramic materials have been reported to exhibit greater resistance to surface alterations induced by toothbrushing, highlighting the relatively higher susceptibility of resin-based materials to mechanical wear. Thus, the null hypothesis was partially rejected.

In terms of color stability, the present study found no statistically significant differences in color change between coated and non-coated groups before and after brushing. These findings suggest that the application of a light-cured nano-filled coating material (Optiglaze™) may contribute to maintaining color stability in 3D-printed provisional crown materials. This observation aligns with previous studies demonstrating that surface coatings can minimize color changes in resin-based materials by forming a smoother, less porous protective layer that reduces pigment adsorption and surface degradation [17]. Within each group, no statistically significant color changes were detected following brushing simulation, indicating that the simulated brushing protocol did not substantially affect the color stability of the tested materials. This outcome may be attributed to the relatively short brushing duration and the smooth surface topography achieved through coating application. Comparable findings were reported by Nakamura *et al*. [18], who observed minimal color alteration in highly polymerized resin surfaces protected by coating layers after toothbrushing simulation. Additionally, Oh *et al*. [16] demonstrated that 3D-printed resin crowns subjected to 50,000 brushing cycles exhibited only minor color changes, confirming the resistance of well-cured and coated printed resins to discoloration under simulated brushing conditions. Collectively, these findings highlight the protective and esthetic benefits of surface coating application, particularly in enhancing color stability and resistance to surface staining.

The results of the present study demonstrated that simulated toothbrushing produced a measurable color change in both the uncoated and OptiGlaze-coated 3D-printed resin specimens. The control group exhibited the lowest color change (ΔE = 1.24), whereas the coated group showed a higher ΔE value (2.1) following brushing simulation. Although the coated specimens demonstrated greater color alteration than the control, the observed ΔE values for both groups remained within clinically acceptable limits, as previously suggested to be limited to 3.3, suggesting that neither condition would result in perceptible or unacceptable esthetic deterioration during clinical service [19].

The slightly higher color change observed in the OptiGlaze-coated group may be attributed to wear or partial degradation of the glaze layer under mechanical brushing forces. Repeated brushing may induce surface abrasion or microcrack formation within the coating, increasing surface irregularities and light scattering, thereby influencing color perception.

The incorporation of surface-modifying agents has been reported to enhance the mechanical properties of dental materials [20,21]. Beyond mechanical performance, surface smoothness plays a critical role in reducing biofilm accumulation on restorative materials [22]. Previous studies have shown that surface roughness exceeding 0.2 µm is associated with increased biofilm adhesion and bacterial retention [23]. In the present study, the application of OptiGlaze as a coating agent effectively reduced surface roughness values and maintained them within clinically acceptable limits even after brushing simulation. This suggests that the OptiGlaze coating may improve surface characteristics.

This study focused only on temporary 3D-printed hybrid resin materials and did not include permanent 3D-printed resins, which may limit the extent to which the results can be applied and the nature of the in vitro study, given current limitations. In addition, the coating thickness and the effect of brushing were not measured or evaluated by scanning electron microscopy after the brushing simulation. The brushing simulation itself was limited to a specific number of cycles and may not reflect long-term clinical use. Future studies should therefore consider increasing the number of brushing cycles to better represent daily oral hygiene over time. The sample size was also relatively small, and using a larger number of specimens could strengthen the reliability of future findings. Moreover, comparing different glazing materials can help determine which offers better surface protection and durability. Future research should also include thermocycling to more closely simulate intraoral aging. Finally, because provisional restorations can remain in the mouth for extended periods, further evaluations, such as exposure to acidic environments and additional mechanical tests, including hardness and flexural strength, are recommended to gain a more complete understanding of material performance.

## Conclusion

This study demonstrated that glazing is a clinically relevant and effective method for enhancing the surface quality of 3D-printed provisional restorative materials. The application of a glaze layer significantly improved surface smoothness without negatively affecting material durability or esthetic stability. Simulated toothbrushing did not produce a statistically significant change in surface roughness in either glazed or non-glazed specimens, indicating stable surface characteristics under oral hygiene conditions. Furthermore, color changes for both groups remained within clinically acceptable limits following brushing simulation, confirming that glazing does not compromise esthetic performance over time. Overall, these findings support glazing as a reliable and practical approach to improving the clinical performance of 3D-printed provisional restorations throughout the temporary treatment period.

## Data Availability

All data used for analysis in the provided study are available at https://doi.org/10.6084/m9.figshare.30818714
